# Enhancement of the thermoelectric properties in bilayer graphene structures induced by Fano resonances

**DOI:** 10.1038/s41598-021-93220-w

**Published:** 2021-07-06

**Authors:** J. A. Briones-Torres, R. Pérez-Álvarez, S. Molina-Valdovinos, I. Rodríguez-Vargas

**Affiliations:** 1 Unidad Académica de Ciencia y Tecnología de la Luz y la Materia, Universidad Autónoma de Zacatecas, Carretera Zacatecas-Guadalajara Km. 6, Ejido La Escondida, 98160 Zacatecas Mexico; 2 Centro de Investigación en Ciencias, Universidad Autónoma del Estado de Morelos, Av. Universidad 1001 Col. Chamilpa, 62209 Cuernavaca, Morelos Mexico

**Keywords:** Thermoelectrics, Two-dimensional materials, Electronic properties and devices

## Abstract

Fano resonances of bilayer graphene could be attractive for thermoelectric devices. The special profile presented by such resonances could significantly enhance the thermoelectric properties. In this work, we study the thermoelectric properties of bilayer graphene single and double barrier structures. The barrier structures are typically supported by a substrate and encapsulated by protecting layers, reducing considerably the phonon thermal transport. So, we will focus on the electronic contribution to the thermal transport. The charge carriers are described as massive chiral particles through an effective Dirac-like Hamiltonian. The Hybrid matrix method and the Landauer–Büttiker formalism are implemented to obtain the transmission, transport and thermoelectric properties. The temperature dependence of the Seebeck coefficient, the power factor, the figure of merit and the efficiency is analyzed for gapless single and double barriers. We find that the charge neutrality point and the system resonances shape the thermoelectric response. In the case of single barriers, the low-temperature thermoelectric response is dominated by the charge neutrality point, while the high-temperature response is determined by the Fano resonances. In the case of double barriers, Breit–Wigner resonances dominate the thermoelectric properties at low temperatures, while Fano and hybrid resonances become preponderant as the temperature rises. The values for the figure of merit are close to two for single barriers and above three for double barriers. The system resonances also allows us to optimize the output power and the efficiency at low and high temperatures. By computing the density of states, we also corroborate that the improvement of the thermoelectric properties is related to the accumulation of electron states. Our findings indicate that bilayer graphene barrier structures can be used to improve the response of thermoelectric devices.

## Introduction

The outstanding properties of two-dimensional (2D) materials make them ideal for plethora of potential applications. In fact, there are already roadmaps for the leading 2D materials^1,2^. In the case of graphene, the high mobility^3,4^ and high thermal conductivity^5,6^ are attractive for thermoelectric and cooling devices. Regarding thermoelectricity, the graphene’s high thermal conductivity is not desirable and several strategies to reduce it have arisen. The main objective of most proposals is to hamper the phonon transport without detriment of the electron transport such that a significant improvement of the figure of merit *ZT* takes place. For instance, in graphene nanoribbons (GNRs) with armchair and zigzag sections arranged in periodic fashion *ZT* attains values above 1^7^. This value for the so-called mixed GNRs is several times higher than the value obtained in pure armchair GNRs. This improvement of *ZT* is a consequence of the significant reduction of the thermal conductance due to the phonon mismatch at the different nanoribbon sections as well as a result of the enhancement of the thermopower due to the resonant tunneling electron transport related to the periodic nanostructuration. $ZTs>2$ are reported in chevron-type^8^, edge-disordered^9^ and extended line defect^10^ GNRs. In all these nanostructures a drastic reduction of the thermal conductance takes place without significant degradation of the electron transport. Ultra-scale structures^11,12^ and anti-dot lattices^13–15^ also provide high values (equal or above 3) of *ZT*. In the former case, the high values are related to a transmission resonance that optimizes the thermopower as well as to the phonon mismatch between the molecule and the GNR leads that reduces significantly the thermal conductance. In the latter case, nanopores degrade substantially the phonon thermal transport and in conjunction with adatoms generate high power factors via edge currents, resulting in outstanding *ZT* values. Another promising structures to achieve high values of *ZT* are the so-called graphene vertical junctions^16–18^. In this structures the weak van-der Waals interaction between graphene layers reduces considerably the phonon thermal transport while the electron transport is weakly affected. Theoretical values above 3 are predicted for *ZT*^16,17^, which are close to the experimental ones found so far^18^. In all these structures the fundamentals of the low-dimensional thermoelectricity are in play^19–21^. In particular, the redistribution of the density of states that enhances the electron transport response and the hamper of the phonon transport that reduces significantly the thermal conductivity. More details about thermoelectric effects in graphene and related 2D materials can be found in the excellent review by Dollfus et al.^22^. Other strategy to improve the thermoelectric response is via substrate engineering^23–25^. This possibility is quite attractive because supporting substrates and encapsulation are essential in the architecture of 2D materials devices. In the case of graphene, the thermal conductivity is significantly reduced due to the deactivation of flexural phonons as a result of supporting and encapsulating the material. There are also important breakthroughs in substrates that do not compromise the electron transport^26–28^. In short, high values of *ZT* are always welcomed because this quantity is directly related to the efficiency $\eta$ of thermoelectric devices. For instance, if $ZT \rightarrow 0$, $\eta \rightarrow 0$, if $ZT \rightarrow \infty$, $\eta \rightarrow \eta _c$, $\eta _c$ being the ideal Carnot efficiency, and if $ZT=1$, $\eta <10$%^29,30^. Hence, the relevance of exceeding the limit $ZT = 1$ to have competitive thermoelectric devices.

Regarding bilayer graphene^31^, it harbors exotic effects that could substantially improve the Seebeck coefficient and the power factor, and consequently giving rise to high values of *ZT*. Among these effects we can find anti-Klein tunneling^32,33^, cloaked states^33,34^, and Fano and hybrid resonances^35–39^. In the case of Fano resonances, it is known that they are related to the chiral nature of electrons in bilayer graphene^33,34,38^. In particular, they arise due to the chiral matching between electron states inside and outside electrostatic barriers at oblique incidence^35–38,40^. On its side, hybrid resonances are the result of the coupling between Fano resonances and resonant states in double and superlattice barrier structures^38,40^. Both types of resonance can retain its special profile if the angle of incidence is restricted to values close to normal incidence^38^. They can also leave a characteristic mark on transport properties that can help to corroborate this exotic phenomenon of Fano resonances in bilayer graphene^38^. Moreover, Fano and hybrid profiles can be manifested in the conductance by opening and modulating a bandgap^41^. Taking into account the special profile of Fano and hybrid resonances as well as its impact on the transport properties of bilayer graphene barrier structures, we can expect high values for the Seebeck coefficient, power factor and *ZT*. Actually, there are several reports in the literature in which is well-documented how Fano resonances enhance thermoelectricity^42–47^. The typical systems consist of quantum-dot interferometers, molecular junctions and chain junctions to mention a few. The common factor in all these studies is the quantum control of Fano resonances to improve the thermoelectric properties. However, as far as we know, there are no reports dealing with the impact of Fano and hybrid resonances on the thermoelectric properties of bilayer graphene barrier structures. In fact, most works about thermoelectricity in bilayer graphene report the Seebeck coefficient in doped systems^48^, systems with applied magnetic fields^49^, disorder^50^, bandgap opening^51^ and strain effects^52–55^. So, we consider that a thorough assessment of the impact of Fano and hybrid resonances on the thermoelectric properties of bilayer graphene barrier structures is necessary.

In the present work, the temperature dependence of the thermoelectric properties of gapless bilayer graphene single and double barriers is studied. We center our study on the thermoelectric response owing to electrons, since it is expected that the phonon thermal transport be reduced considerably due to the substrate and encapsulating layers typical of barrier structure devices^23–25^. The charge carriers in bilayer graphene are described as quantum relativistic particles through an effective Dirac-like Hamiltonian. The hybrid matrix method is used to calculate the transmission probability or transmittance^40,56,57^, while the Landauer–Büttiker formalisms is implemented to obtain the conductance, Seebeck coefficient, power factor, *ZT* and $\eta$^58,59^. The impact of the system resonances on the thermoelectric properties is analyzed. It is found that at low-temperatures the charge neutrality point and the Breit–Wigner resonances dominate the thermoelectric response and at high-temperatures the Fano and hybrid resonances become preponderant.

## Methodology

**Figure 1:**
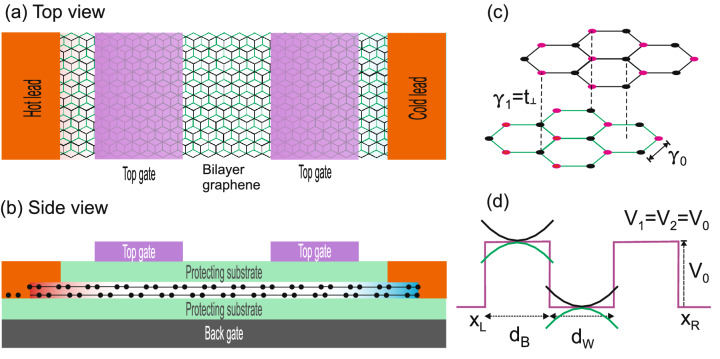
(**a**) Top and (**b**) side view of the schematic representation of the possible thermoelectric device based on bilayer graphene double barriers. The double barrier structure generated by the top gates is sandwiched between hot and cold leads. (**c**) Crystal structure of Bernal-stacked bilayer graphene, with $\gamma _{0}$ and $\gamma _{1}= t_{\perp }$ as the main interactions between carbon atoms. (**d**) Band-edge profile of the conduction band of (**a**). Here, $V_{0}=V_{1}=V_{2}$ represent the potential energy on the bilayer graphene layers, respectively, also the potentials $V_{1}$ and $V_{2}$ are less than $t_{\perp }$ and therefore the bands can be considered as paraboloids. $d_{B}$ and $d_{w}$ represents the widths of the barrier and well regions, respectively. $x_L$ and $x_R$ correspond to the left and right ends of the nanostructure.

In Fig. 1a, b we illustrate the possible thermoelectric device based on bilayer graphene barrier structures. As a representative case we are showing a double-barrier thermoelectric device. The heat flows from the left to the right lead, hot and cold side, respectively. Bilayer graphene is placed on a substrate, which in turn is located above a back gate. The potential barriers are generated by electrodes (gates) on top of bilayer graphene. The crystal structure is schematically shown in Fig. 1c. It corresponds to stable Bernal-stacked bilayer graphene. The relevant interactions between carbon atoms for our Hamiltonian are depicted as well, $\gamma _{0}$ and $\gamma _{1}=t_{\perp }$. The potential barriers are illustrated in Fig. 1d. They correspond to gapless barriers, that is, the potential energy is the same in both graphene layers $V_1=V_2=V_0$. It is also important to mention that as graphene is supported by a substrate and typically encapsulated with dielectrics, we expect that phonon contribution to the thermal transport be negligible^23–25^. So, we will only consider the contribution of electrons to the thermal transport.

To study the thermoelectric properties of bilayer graphene structures we will use the stable numerical method known as the hybrid matrix method^40,56,57^. In this problem, we deal with a system of first-order ordinary differential equations. To use transfer matrix techniques, and in particular the so-called hybrid matrices, we have made the necessary adjustments, which on the other hand are quite obvious and are explained elsewhere^40,41,57^. To establish the hybrid matrix method, we need firstly the effective Dirac Hamiltonian that describes the charge carriers in bilayer graphene, namely^60–62^:

1 $$\begin{aligned} \mathcal {H}=\left( \begin{array}{cccc} V_{1}&{}\pi &{}t_{\perp }&{}0\\ \pi ^{*}&{}V_{1}&{}0&{}0\\ t_{\perp }&{}0&{}V_{2}&{}\pi ^{*}\\ 0&{}0&{}\pi &{}V_{2} \end{array} \right) , \end{aligned}$$

where $\pi =v_{F}(p_{x}+ip_{y})$, $\pi ^{*}=v_{F}(p_{x}-ip_{y})$, $p_{x,y}=-i\hbar \partial _{x,y}$ is the momentum operator and $v_{F}$ is the Fermi velocity. This Hamiltonian is valid for energies less than $\gamma _{0}=3.09$ eV^60,61^, it involves the interaction between graphene layers through the parameter $t_{\perp }=390$ meV^61^ and takes into account bandgap opening $E_{g}=V_{2}-V_{1}$ when $V_1 \ne V_2$.

Now, it is important to mention that in our single and double barrier structures there is spatial homogeneity in the transverse direction, that is $q_{y}=k_{y}$, then the propagation of Dirac electrons can be reduced to a one-dimensional problem. So, we need secondly to solve the one-dimensional eigenvalue equation $\mathbf {H}\cdot \mathbf {F}(x)=E\mathbf {F}(x)$, which after basic algebra adopts the mathematical form^41^

2 $$\begin{aligned} \frac{d\mathbf {F}(x)}{dx}+\left( \begin{array}{cccc} q_{y}&{}i\frac{V_{1}-E}{\hbar v_{F}}&{}0&{}0\\ i\frac{V_{1}-E}{\hbar v_{F}}&{}-q_{y}&{}i\frac{t_{\perp }}{\hbar v_{F}}&{}0\\ 0&{}0&{}-q_{y}&{}i\frac{V_{2}-E}{\hbar v_{F}} \\ i\frac{t_{\perp }}{\hbar v_{F}}&{}0&{}i\frac{V_{2}-E}{\hbar v_{F}}&{}q_{y} \end{array}\right) \cdot \mathbf {F}(x)=\mathbf {0}_{4\times 1}. \end{aligned}$$

So, to find a basis, a function of the form $\mathbf {F}(x)=\mathbf {F}_{0}e^{iqx}$ is proposed, this is substituted in Eq. (2) to arrive at a secular equation that has the following eigenvalues as solution

3 $$\begin{aligned} q=\pm \sqrt{-q^{2}_{y}+\frac{1}{2(\hbar v_{F})^{2}}[(E-V_{1})^{2}+(E-V_{2})^{2}]\pm \frac{1}{2(\hbar v_{F})^{2}}\sqrt{[(E-V_{1})^{2}-(E-V_{2})^{2}]^{2}+4t^{2}_{\perp }(E-V_{1})(E-V_{2})}}. \end{aligned}$$

We can see that there are four eigenvalues, this comes from the fact that the Hamiltonian is 4$\times$4, then we have four linear independent solutions given as

4 $$\begin{aligned} \mathbf {F}(x)^{\pm }_{j}=\mathbf {F}_{0j}^{\pm }e^{\pm iq_{j}x} = (a_{j},b_{j}^{\pm },c_{j},d_{j}^{\pm })^{T}e^{\pm iq_{j}x},\quad j=1,2. \end{aligned}$$

where the corresponding components are

5 $$\begin{aligned} a_{j}= & {} 1; \end{aligned}$$

6 $$\begin{aligned} b_{j}^{\pm }= & {} \frac{\hbar v_{F}(\pm q_{j}-iq_{y})}{E-V_{1}};\end{aligned}$$

7 $$\begin{aligned} c_{j}= & {} \frac{t_{\perp }(E-V_{2})}{(E-V_{2})^{2}-(\hbar v_{F})^{2}(q_{j}^{2}+q_{y}^{2})};\end{aligned}$$

8 $$\begin{aligned} d_{j}^{\pm }= & {} \frac{\hbar v_{F}(\pm q_{j}+iq_{y})}{E-V_{2}}c_{j}. \end{aligned}$$

A general solution can be expressed in matrix form as follows

9 $$\begin{aligned} \mathbf {F}(x) = \left( \begin{array}{cccc} a_{1}e^{iq_{1}x}&{}a_{2}e^{iq_{2}x}&{}a_{1}e^{-iq_{1}x}&{}a_{2}e^{-iq_{2}x}\\ b_{1}^{+}e^{iq_{1}x}&{}b_{2}^{+}e^{iq_{2}x}&{}b_{1}^{-}e^{-iq_{1}x}&{}b_{2}^{-}e^{-iq_{2}x}\\ c_{1}e^{iq_{1}x}&{}c_{2}e^{iq_{2}x}&{}c_{1}e^{-iq_{1}x}&{}c_{2}e^{-iq_{2}x}\\ d_{1}^{+}e^{iq_{1}x}&{}d_{2}^{+}e^{iq_{2}x}&{}d_{1}^{-}e^{-iq_{1}x}&{}d_{2}^{-}e^{-iq_{2}x} \end{array}\right) \cdot \left( \begin{array}{c} \alpha _{1}^{+}\\ \alpha _{2}^{+}\\ \alpha _{1}^{-}\\ \alpha _{2}^{-} \end{array}\right) \end{aligned}$$

or equivalently by matrix blocks

10 $$\begin{aligned} \mathbf {F}(x)=\left( \begin{array}{c} \mathbf {F}_{u}(x)\\ \mathbf {F}_{d}(x) \end{array}\right) =\left( \begin{array}{cc} \mathbf {U}^{+}(x) &{} \mathbf {U}^{-}(x)\\ \mathbf {D}^{+}(x) &{} \mathbf {D}^{-}(x) \end{array}\right) \cdot \left( \begin{array}{c} \alpha ^{+}\\ \alpha ^{-} \end{array}\right) , \end{aligned}$$

where $\mathbf {F}_{u}(x)$, $\mathbf {F}_{d}(x)$, $\alpha ^{+}$ and $\alpha ^{-}$ are a two-dimensional column vectors. $\mathbf {U}^{\pm }(x)$ and $\mathbf {D}^{\pm }(x)$ are the $2\times 2$ respective matrix blocks of the $4\times 4$ matrix in equation (9). We can define the hybrid matrix as

11 $$\begin{aligned} \left( \begin{array}{c} \mathbf {F}_{u}(x_{L})\\ \mathbf {F}_{d}(x_{R}) \end{array}\right) =\mathbf {H}(x_{R},x_{L})\cdot \left( \begin{array}{c} \mathbf {F}_{d}(x_{L})\\ \mathbf {F}_{u}(x_{R}) \end{array}\right) . \end{aligned}$$

This equation relates the vectors $\mathbf {F}_{u}(x)$ and $\mathbf {F}_{d}(x)$ at the left ($x_{L}$) and right ($x_{R}$) ends of the heterostructure, see Fig. 1d. The explicit expressions for the vectors $\mathbf {F}_{u}(x)$ and $\mathbf {F}_{d}(x)$ can be found in Ref. 41. Then from equations (10) and (11), the hybrid matrix can be written as

12 $$\begin{aligned} \mathbf {H}(x_{R},x_{L})= & {} \left( \begin{array}{cccc} a_{1}e^{iq_{1}x_{L}}&{}a_{2}e^{iq_{2}x_{L}}&{}a_{1}e^{-iq_{1}x_{L}}&{}a_{2}e^{-iq_{2}x_{L}}\\ b_{1}^{+}e^{iq_{1}x_{L}}&{}b_{2}^{+}e^{iq_{2}x_{L}}&{}b_{1}^{-}e^{-iq_{1}x_{L}}&{}b_{2}^{-}e^{-iq_{2}x_{L}}\\ c_{1}e^{iq_{1}(x_{R})}&{}c_{2}e^{iq_{2}(x_{R})}&{}c_{1}e^{-iq_{1}(x_{R})}&{}c_{2}e^{-iq_{2}(x_{R})}\\ d_{1}^{+}e^{iq_{1}(x_{R})}&{}d_{2}^{+}e^{iq_{2}(x_{R})}&{}d_{1}^{-}e^{-iq_{1}(x_{R})}&{}d_{2}^{-}e^{-iq_{2}(x_{R})} \end{array}\right) \nonumber \\&\cdot \left( \begin{array}{cccc} c_{1}e^{iq_{1}x_{L}}&{}c_{2}e^{iq_{2}x_{L}}&{}c_{1}e^{-iq_{1}x_{L}}&{}c_{2}e^{-iq_{2}x_{L}}\\ d_{1}^{+}e^{iq_{1}x_{L}}&{}d_{2}^{+}e^{iq_{2}x_{L}}&{}d_{1}^{-}e^{-iq_{1}x_{L}}&{}d_{2}^{-}e^{-iq_{2}x_{L}}\\ a_{1}e^{iq_{1}(x_{R})}&{}a_{2}e^{iq_{2}(x_{R})}&{}a_{1}e^{-iq_{1}(x_{R})}&{}a_{2}e^{-iq_{2}(x_{R})}\\ b_{1}^{+}e^{iq_{1}(x_{R})}&{}b_{2}^{+}e^{iq_{2}(x_{R})}&{}b_{1}^{-}e^{-iq_{1}(x_{R})}&{}b_{2}^{-}e^{-iq_{2}(x_{R})} \end{array}\right) ^{-1}. \end{aligned}$$

Equation (12) is the hybrid matrix for a homogeneous domain, in our case well or barrier. To obtain the hybrid matrix for a heterostructure it is essential to know the composition rule, for more details see^38,40,56^. Then, in order to know the transmission and transport properties of a heterostructure it is necessary to know the vectors $\mathbf {F}_{u}(x)$ and $\mathbf {F}_{d}(x)$ of Eq. (10) at the left ($x_{L}$) and right ($x_{R}$) ends of the heterostructure and the total hybrid matrix, see Ref. 41.

We assume that a wave $\mathbf {F}^{+}_{01}e^{iq_{1}x}$ traveling from the left side hits the left end of the barrier structure and results in reflections $\mathbf {F}^{-}_{01}e^{-iq_{1}x}$ and $\mathbf {F}^{-}_{02}e^{-iq_{2}x}$ in that domain, while at the right end of the barrier structure we have only transmitted waves $\mathbf {F}^{+}_{01}e^{iq_{1}x}$ and $\mathbf {F}^{+}_{02}e^{iq_{2}x}$, this is apparently a multi-channel problem, but on both the left and right side we have non-propagating solutions. This makes us really face a problem with a single channel. Then taking into account the above, equation (11) takes the form

13 $$\begin{aligned} \mathbf {M}_{1}+\mathbf {M}_{2}\cdot \left( \begin{array}{c} r_{1}\\ r_{2}\\ t_{1}\\ t_{2} \end{array}\right) = \mathbf {H}(x_{R},x_{L})\cdot \mathbf {M}_{3}+\mathbf {H}(x_{R},x_{L})\cdot \mathbf {M}_{4}\cdot \left( \begin{array}{c} r_{1}\\ r_{2}\\ t_{1}\\ t_{2} \end{array}\right) , \end{aligned}$$

where $r_{1}, r_{2}, t_{1}$ and $t_{2}$ are defined in terms of the coefficients $\alpha ^{+}$ and $\alpha ^{-}$ at each end^41^. In addition, the matrices $\mathbf {M}_{1},\mathbf {M}_{2}, \mathbf {M}_{3}$ and $\mathbf {M}_{4}$ are given as

14 $$\begin{aligned} \mathbf {M}_{1}= & {} \left( \begin{array}{c} a_{1L}\\ b^{+}_{1L}\\ 0\\ 0 \end{array} \right) ,\quad \mathbf {M}_{2}=\left( \begin{array}{cccc} a_{1L}&{}a_{2L}&{}0&{}0\\ b^{-}_{1L}&{}b^{-}_{2L}&{}0&{}0\\ 0&{}0&{}c_{1R}&{}c_{2R}\\ 0&{}0&{}d^{+}_{1R}&{}c^{+}_{2R} \end{array} \right) , \end{aligned}$$

15 $$\begin{aligned} \mathbf {M}_{3}= & {} \left( \begin{array}{c} c_{1L}\\ d^{+}_{1L}\\ 0\\ 0 \end{array} \right) ;\quad \mathbf {M}_{4}=\left( \begin{array}{cccc} c_{1L}&{}c_{2L}&{}0&{}0\\ d^{-}_{1L}&{}d^{-}_{2L}&{}0&{}0\\ 0&{}0&{}a_{1R}&{}a_{2R}\\ 0&{}0&{}b^{+}_{1R}&{}b^{+}_{2R} \end{array} \right) . \end{aligned}$$

The subscripts *L* and *R* indicate the external domain where the components of the wavefunction amplitudes are calculated. Finally, the transmission and reflection amplitudes can be written in terms of the hybrid matrix in a more compact form as

16 $$\begin{aligned} \left( \begin{array}{c} r_{1}\\ r_{2}\\ t_{1}\\ t_{2} \end{array}\right) = \left[ \mathbf {M}_{2}-\mathbf {H}(x_{R},x_{L})\cdot \mathbf {M}_{4}\right] ^{-1} \cdot \left[ \mathbf {H}(x_{R},x_{L})\cdot \mathbf {M}_{3}-\mathbf {M}_{1}\right] . \end{aligned}$$

In general, we are dealing with a problem of two transmission channels. So, the transmittance will be given by

17 $$\begin{aligned} \mathbb {T}(E,\theta )=\left| t_{1}\right| ^2+\left| t_{2}\right| ^2, \end{aligned}$$

where *E* and $\theta$ are the energy and angle of incidence of the electrons in the bilayer graphene structure.

However, if $E< t_{\perp }$ there is only one transmission channel, as can be seen from Eq. (3) at the semi-infinite regions $V_1=V_2=0$. In such case, the transmittance is simply

18 $$\begin{aligned} \mathbb {T}(E,\theta )=\left| t_{1}\right| ^2. \end{aligned}$$

In fact, in all our calculations the one transmission channel condition $E< t_{\perp }$ is fulfilled. Thus, the transmittance that we will use is determined by Eq. (18).

To calculate the transport and thermoelectric properties it is necessary to obtain the conductance function, this can be done using the Landauer–Büttiker formalism^58,59^. Under this formalism, the conductance function can be obtained by summing the transmittance over all angles of incidence

19 $$\begin{aligned} {G}(E)=G_{0}\sqrt{E^{2}+t_{\perp }E}\int _{-\frac{\pi }{2}}^{\frac{\pi }{2}}\mathbb {T}(E,\theta )\cos {\theta } d\theta , \end{aligned}$$

where $G_{0}=\frac{2e^{2}L_{y}}{h^{2}v_{F}}$ is the fundamental conductance factor with *e*, $L_{y}$ and *h* as the electron charge, the width of bilayer graphene sheet in the transverse *y*-coordinate and the Planck’s constant, respectively. For our calculations, the width of the bilayer graphene is set to $L_{y} = 200$ nm^63^.

In the case of thermoelectric properties, it is necessary to know the Seebeck coefficient, it can be done with the help of Landauer–Büttiker formalism as well^59,64^. If we consider an effective voltage $\Delta \mathcal {V}=\mathcal {V}_1-\mathcal {V}_2$ and a temperature gradient $\Delta T=T_1-T_2$ between the source and the drain in Fig. 1a, the linear-regime current of electrons in the system can be written as

20 $$\begin{aligned} I = G {\Delta \mathcal {V}} + G_{S}\Delta T, \end{aligned}$$

where the conductance is given by

21 $$\begin{aligned} G(\mu )=\int _{-\infty }^{\infty }G(E)\left( -\frac{\partial f_{0}}{\partial E}\right) dE, \end{aligned}$$

and the $G_{S}$ coefficient by

22 $$\begin{aligned} G_{S}(\mu )=\int _{-\infty }^{\infty }G(E)\frac{E-\mu }{eT}\left( -\frac{\partial f_{0}}{\partial E}\right) dE, \end{aligned}$$

with *T*, $\mu$, and $f_{0}$ as the average temperature, the average chemical potential and Fermi-Dirac distribution function, respectively. Then the Seebeck coefficient is defined as the voltage induced by a temperature gradient in open circuit condition $(I=0)$, namely,

23 $$\begin{aligned} S = -\frac{{\Delta \mathcal {V}}}{\Delta T} = \frac{G_{S}}{G}. \end{aligned}$$

Moreover, to characterize the thermoelectric performance of a device we need to consider *ZT*, which is nothing other than the amount of electricity that can be generated from a temperature gradient. Specifically, *ZT* is given by the expression

24 $$\begin{aligned} ZT = \frac{S^{2}GT}{G_{K}}. \end{aligned}$$

Here, the quantity $S^{2}G$ is known as the power factor, for a material to be a good thermoelectric, its power factor needs to be as high as possible. $G_K$ is the so-called electronic thermal conductance at open circuit ($I=0$), which needs to be as low as possible for a high *ZT*. $G_{K}$ is defined by the thermal current

25 $$\begin{aligned} I_{Q} = \frac{G_{P}}{G} I + G_{K}\Delta T, \end{aligned}$$

and given as

26 $$\begin{aligned} G_{K} = -\frac{I_{Q}}{\Delta T} = G_{Q}-\frac{G_{P}G_{S}}{G}, \end{aligned}$$

where

27 $$\begin{aligned} G_{P}(\mu )=\int _{-\infty }^{\infty }G(E)\frac{E-\mu }{e}\left( -\frac{\partial f_{0}}{\partial E}\right) dE \end{aligned}$$

and

28 $$\begin{aligned} G_{Q}(\mu )=\int _{-\infty }^{\infty }G(E)\frac{(E-\mu )^{2}}{e^{2}T}\left( -\frac{\partial f_{0}}{\partial E}\right) dE \end{aligned}$$

are the Peltier transport coefficient and the thermal conductance at short circuit (${\Delta \mathcal {V}}=0$), respectively. To end the mathematics of the thermoelectric properties, we would like to mention that to compute the transport coefficients *G*, $G_{S}$, $G_{P}$, $G_{K}$ and $G_{Q}$ it is sufficient to consider a few $k_{B}T$ around $\mu$ due to the broadening function $\left( -\frac{\partial f_{0}}{\partial E}\right)$ and not the entire energy range.

With the transport coefficients we can also compute straightforwardly the maximum power^54^

29 $$\begin{aligned} P^{max}=\frac{1}{4} S^2 G \left( \Delta T\right) ^2 \end{aligned}$$

and the efficiency at maximum power^54^

30 $$\begin{aligned} \eta (P^{max})=\frac{\eta _c}{2} \frac{ZT}{2+ZT}. \end{aligned}$$

To end this section, we will give the fundamentals to determine the density of states (DOS) of single and double barriers. This calculation will give us the possibility of know if the accumulation of electron states is related to the improvement of the thermoelectric response. The DOS can be obtained straightforwardly through the system band structure. In the case of one-dimensional periodic system the DOS per unit length and energy is given as^59^

31 $$\begin{aligned} \text {DOS}(E,\theta ) = \frac{1}{2\pi }\left| \frac{\partial q_{Bl}(E,\theta )}{\partial E}\right| , \end{aligned}$$

where $q_{Bl}$ is the Bloch wave vector associated to the super-periodicity. Here, we have also remarked that in a one dimensional periodic system in 2D materials there is a dependence on the transverse wave vector or equivalently on the angle of incidence. In fact, we can obtain the total DOS by summing over all angles of incidence, namely:

32 $$\begin{aligned} \text {DOS}(E)=\frac{L_{y}}{2\pi }\frac{\sqrt{E^{2}+t_{\perp }E}}{\hbar v_{F}}\int _{-\frac{\pi }{2}}^{\frac{\pi }{2}}\text {DOS}(E,\theta )\cos {\theta } d\theta . \end{aligned}$$

Up to this point, the fundamentals to compute the DOS are quite clear, however we are not dealing with periodic or superlattice structures. To overcome this detail, we will consider single barriers as a periodic system with a unit-cell compose of a barrier and a well. Then, we will try to mimic single barriers by increasing systematically the width of the well region. In the case of double barriers, we will consider as unit-cell two barriers and two wells, and the width of the second well will be systematically increased to mimic double barriers. By applying the Bloch’s theorem to the mentioned periodic systems of single and double barriers we can obtain its corresponding band structure^61^. Specifically, from the Bloch condition

33 $$\begin{aligned} \mathbf {F}(x+d_{uc})=e^{iq_{Bl}d_{uc}} \mathbf {F}(x), \end{aligned}$$

we can derive the following transcendental equation

34 $$\begin{aligned} \text {det}\left( \prod _{j=1}^{N}\left( D_j\cdot P_j^{-1}\cdot D_j^{-1}\right) -e^{iq_{Bl}d_{uc}}I\right) =0, \end{aligned}$$

where $d_{uc}$ is the size of the unit-cell, and $D_j$ and $P_j$ are matrices given in terms of the coefficients and wave vectors of the wave functions of the barrier and well regions that compose the unit-cell, namely:

35 $$\begin{aligned} D_{j}= & {} \left( \begin{array}{cccc} a_{1,j} &{} a_{2,j} &{} a_{1,j} &{} a_{2,j} \\ b^{+}_{1,j} &{} b^{+}_{2,j} &{} b^{-}_{1,j} &{} b^{-}_{2,j} \\ c_{1,j} &{} c_{2,j} &{} c_{1,j} &{} c_{2,j} \\ d^{+}_{1,j} &{} d^{+}_{2,j} &{} d^{-}_{1,j} &{} d^{-}_{2,j} \end{array} \right) ; \end{aligned}$$

36 $$\begin{aligned} P_{j}^{-1}= & {} \left( \begin{array}{cccc} e^{iq_{1,j}\;d_j} &{} 0 &{} 0 &{} 0 \\ 0 &{} e^{iq_{2,j}\;d_j} &{} 0 &{}0 \\ 0 &{} 0 &{} e^{-iq_{1,j}\;d_j} &{} 0 \\ 0 &{} 0 &{} 0 &{} e^{-iq_{2,j}\;d_j} \end{array} \right) . \end{aligned}$$

Here, $j=1,3$ and $j=2,4$ correspond to barrier and well regions respectively. Furthermore, if $N = 2$, we are talking about a unit cell formed by a barrier plus a well with $d_{uc} =d_B+d_W$. For the case of two barriers (N = 4), the unit cell is composed of two barriers and two wells with $d_{uc} = d_{B_1}+d_{W_1}+d_{B_2}+d_{W_2}$. Finally, with the dispersion relation $E=E(q_{Bl})$, we can obtain the *DOS*(*E*) straightforwardly by using Eqs. (31) and (32).

## Results and discussion

As we have documented resonances can improve the thermoelectric properties. In fact, the so-called Mahan-Sofo criteria tell us that a resonance, its shape and its width play a crucial role in the thermoelectric response^65,66^. This is quite relevant in the case of bilayer graphene single and double barriers owe Breit–Wigner, Fano and hybrid resonances arise in the transmission properties^38^. Moreover, these resonances leave an identifiable characteristic mark on transport properties^38^. In view of this, it is essential to analyze whether thermoelectric properties improve or perhaps exceed current reported values. The thermoelectric properties in our case are calculated using the Landauer–Büttiker formalism^58,59^, which implies the computation of the integrals of the transport coefficients *G*, $G_S$, $G_P$ and $G_Q$, expecting that the Breit–Wiger, Fano and hybrid resonances play a preponderant role. To have reliable results for the transport coefficients we have considered up to 20 $k_BT$ around $\mu$ in the integrals.

Under this context, we firstly show the results for the conductance function, the Seebeck coefficient, the power factor and *ZT* of gapless bilayer graphene single barriers. Secondly, we analyze the corresponding results of gapless bilayer graphene double barriers. We paid special attention to the impact of the system resonances on the mentioned thermoelectric properties.

### Bilayer graphene single barriers

**Figure 2:**
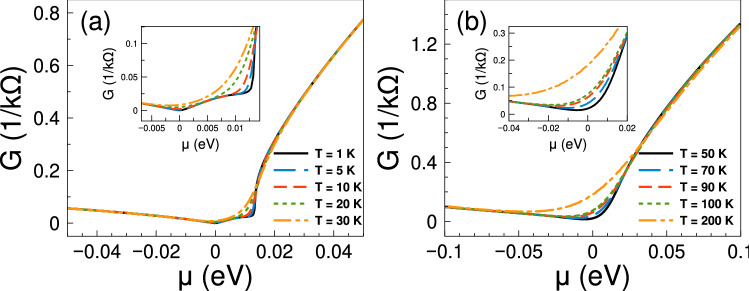
Conductance function versus chemical potential for gapless bilayer graphene single barriers at different temperatures: (**a**) from 1 to 30 K and (**b**) from 50 to 200 K. The single barrier structural parameters, width and height of the barriers, are $d_{B}=6$ nm and $V_{0}=50$ meV, respectively. The insets show a zoom of the conductance at low chemical potentials.

At first place, we consider the transport and thermoelectrics of single barriers. Here and henceforth, we will consider gapless barriers, that is, barriers in which $V_1=V_2=V_0$. We will begin by analyzing the energy regions where Fano resonances occur in electron transport. Figure 2 shows the conductance as a function of chemical potential at: (a) low temperatures and (b) high temperatures. At low temperatures the conductance shows a markedly sudden rise around 12.5 meV, which is associated to the Fano resonances of the system^38^. As the temperature grows the sudden rise softens and increases monotonically. Furthermore, we can notice that the charge neutrality point is well-defined at low temperatures, that is, there is a change of slope in the conductance between electrons and holes. As the temperature rises the change of slope is reduced, however it still persists at high temperatures. All these characteristics will have a direct impact in the thermoelectric properties.

**Figure 3:**
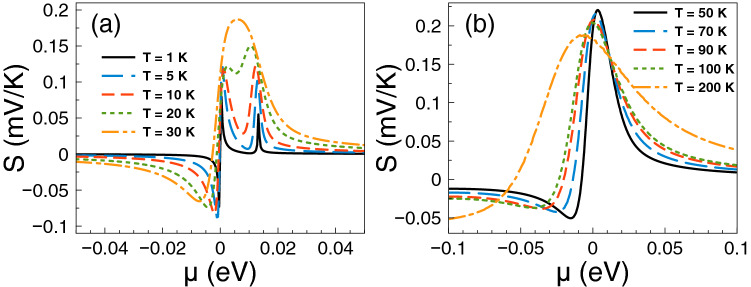
The Seebeck coefficient versus chemical potential for gapless bilayer graphene single barriers at different temperatures: (a) from 1 K to 30 K and (b) from 50 K to 200 K. The single barrier structural parameters are the same as in Fig. 2. As it is noted the charge neutrality point shapes the Seebeck coefficient characteristics at low temperatures, while the Fano resonances region become preponderant as the temperature rises.

The contribution of the Fano resonances to the thermoelectric properties, particularly to the Seebeck coefficient, is shown in Fig. 3. The same parameters as in Fig. 2 are considered. As we can see the Seebeck coefficient shows a pair of peaks in the electron region, one due to the charge neutrality point and the other due to the Fano resonances. At temperatures from 1 to 10 K the charge neutrality point dominates over the Fano resonances, then they combine in a single peak at T = 30 K with its maximum at the charge neutrality point. However, as the temperature increases the peak reaches a maximum of 250 $\mu$V/K at T = 50 K around 12.5 meV, where the Fano resonances take place. By further increasing the temperature the peak widens and decreases systematically. We can also see a peak in the hole branch that is affected by temperature effects. This peak is even higher at low temperatures than the one associated to Fano resonances, however as the temperature rises the peaks in the electron branch dominate the Seebeck coefficient characteristics.

**Figure 4:**
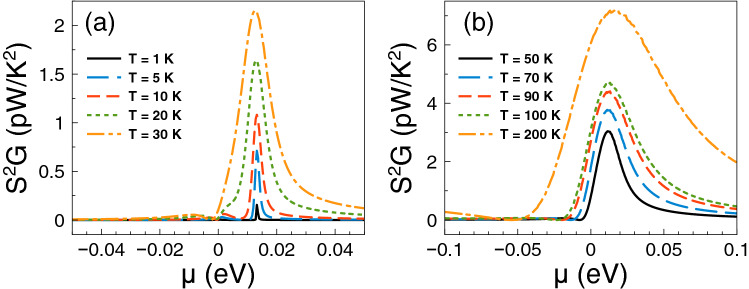
Power factor versus chemical potential for gapless bilayer graphene single barriers at different temperatures: (**a**) from 1 to 30 K and (**b**) from 50 to 200 K. The single barrier structural parameters are the same as in Fig. 2. In this case, the Fano resonances energy region dominates the power factor characteristics at practically all temperatures.

Having the conductance and the Seebeck coefficient we can obtain straightforwardly the power factor $S^{2}G$. In fact, the corresponding results of the power factor are shown in Fig. 4. As we can see the power factor is maximized in the energy regions in which the Fano resonances are preponderant. For practically all temperatures the contribution of the Fano resonances dominates the power factor characteristics. This is to be expected since the power factor is the result of the interplay between the square of the Seebeck coefficient and the conductance. So, it is not sufficient to have a high Seebeck coefficient to obtain a high power factor, likewise, it is not enough to have a large conductance to obtain a high power factor. Actually, it is the combination of both, the Seebeck and the conductance, that shapes the power factor. In the present case, the combination is enhanced precisely in the energy region in which Fano resonances are preponderant, in great extent due to the sudden rise presented in the conductance as well as the associated peak in the Seebeck coefficient. Furthermore, the increase in the power factor is monotonous with respect to temperature and, as in the case of the Seebeck coefficient, the peak is sharp at low temperatures and widens at high temperatures.

**Figure 5:**
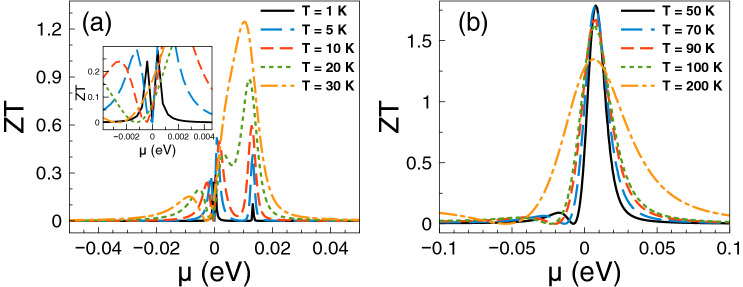
*ZT* versus chemical potential for gapless bilayer graphene single barriers at different temperatures: (**a**) from 1 to 30 K and (**b**) from 50 to 200 K. The single barrier structural parameters are the same as in the preceding figures. As in the case of the Seebeck coefficient, the charge neutrality point dominates the *ZT* characteristics at low temperatures, while the Fano resonances energy region become preponderant as the temperature rises.

**Figure 6:**
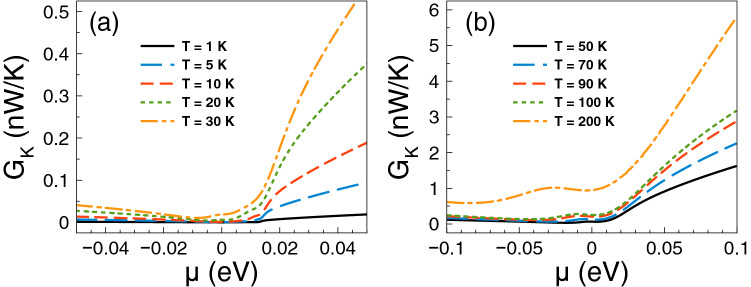
Electronic thermal conductance versus chemical potential for gapless bilayer graphene single barriers at different temperatures: (**a**) from 1 to 30 K and (**b**) from 50 to 200 K. The single barrier structural parameters are the same as in the preceding figures. The electronic thermal conductance is similar to the electrical conductance. However, the ratio of $G_K$ to *GT* is not at all the Lorenz number, resulting in values of *ZT* an order of magnitude lower than in strained single layer graphene^54^. More details about $G_K/(GT)$ can be found in the supplementary information.

**Figure 7:**
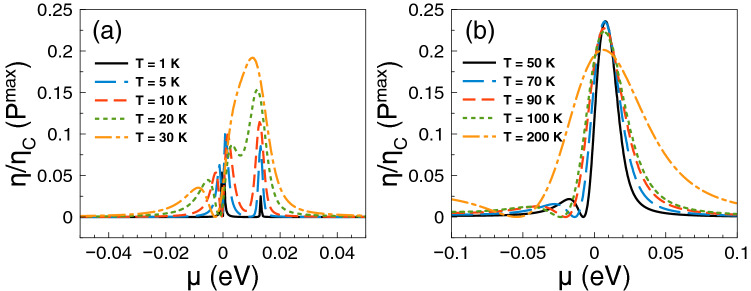
Efficiency at maximum power versus chemical potential for gapless bilayer graphene single barriers at different temperatures: (**a**) from 1 to 30 K and (**b**) from 50 to 200 K. The single barrier structural parameters are the same as in the preceding figures. The maximum efficiency is reached at the temperatures at which *ZT* is maximum as well.

Regarding *ZT* it shows a behavior similar to that of the Seebeck coefficient and a monotonous growth as of the power factor. The *ZT* results are shown in Fig. 5. As we can notice at low temperatures, from 1 to 10 K, the charge neutrality point dominates, while from 20 K onwards Fano resonances shape the *ZT* characteristics. The maximum value reached by *ZT* is 1.8 at 50 K. For temperatures above 50 K, *ZT* decreases monotonically and the peak associated to Fano resonances broadens systematically as well. At first instance the behavior of *ZT* seems counterintuitive because there is no such competition between the charge neutrality point and the Fano resonances in the power factor results. However, for *ZT* we cannot forget the contribution of the electronic thermal conductance. The specific results for this important quantity are shown in Fig. 6. As we can see the general dependence of the thermal conductance is similar to the corresponding one presented by the electrical conductance. Specifically, it has a significant rise at the Fano resonances energy region and a systematic increase with temperature. In fact, this similarity between both conductances is what makes that the Seebeck coefficient shapes the *ZT* characteristics. However, the *ZT* values are not as high as in the case of strained single layer graphene with a spin flipper^54^, because $G_K/(GT)$ is not proportional to the Lorenz number in the entire chemical potential energy range. Actually, this quantity attains large values in the energy regions in which $S^2$ is large as well, resulting in values of *ZT* up to 1.7. The results of $G_K/(GT)$ and $S^2$ as a function of the chemical potential for different temperatures can be found in the supplementary information. In addition, the comparison of the thermometric response of bilayer graphene with and without barriers is presented in the supplementary information. From this comparison, we can realize that the effects of the barriers are fundamental to improve the thermoelectric response. To end with the thermoelectric response of single barriers, we will analyze the results for the maximum power and the efficiency at maximum power. Regarding the former, the results are essentially the same as in Fig. 4, it is just a matter of weigh them by the factor 1/4 and $(\Delta T)^2$. For instance, the maximum power at $T=30$ K is 0.5387 pW$(\Delta T)^2$/K$^2$, while at $T=100$ K is 1.755 pW$(\Delta T)^2$/K$^2$. These values are more than an order of magnitude higher than the ones reported in strained single layer graphene^54^. In fact, the maximum power of single barriers is a monotonous increasing function of the temperature. In addition, the peak of the maximum power shifts to higher chemical potentials as the temperature rises. This would be welcomed if the efficiency would have the same pace, however, in general this is not the case. In the present case of single barriers the maximum efficiency is reached at $T=50$ K and $\mu \approx 15$ meV as shown in Fig. 7. Actually, we consider that these parameter are the optimum in the case of single barriers with $P^{max}=0.75$ pW$(\Delta T)^2$/K$^2$ and $\eta =0.23\eta _c$.

**Figure 8:**
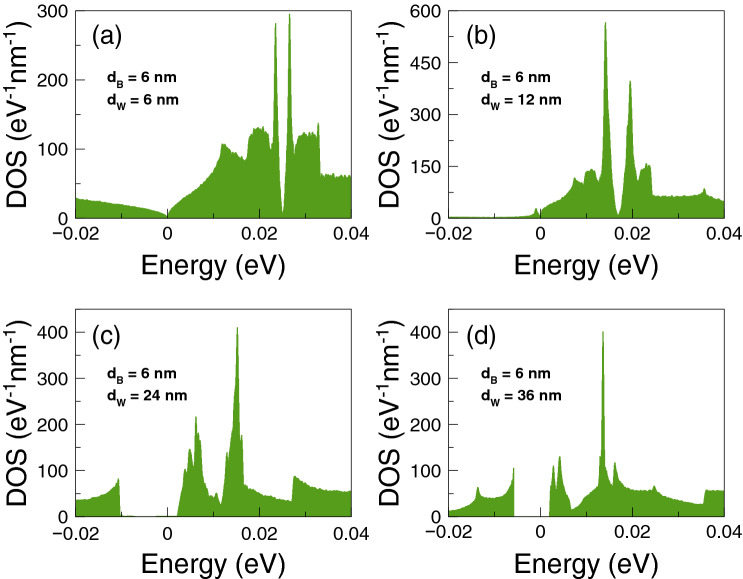
Density of states versus the energy for gapless bilayer graphene single barriers of $d_{B}=6$ nm. Here, the width of the well region $d_{W}$ is varied to mimic the conditions for single barriers: (**a**) $d_W=6$ nm, (**b**) $d_W=12$ nm, (**c**) $d_W=24$ nm and (**d**) $d_W=36$ nm. The height of the barrier is $V_{0}=50$ meV. As $d_W$ increases the electron states are accumulated around the energy region in which the Fano resonances are preponderant.

Finally, we will analyze if the accumulation of the DOS is related to the improvement of the thermoelectric properties. The DOS outcomes as function of the energy are shown in Fig. 8. We have considered one-dimensional periodic structures with a barrier and a well as unit-cell. The width of the well region is varied in order to mimic the conditions for single barriers, that is, a barrier with left and right semi-infinite regions. Specifically, we have considered well widths of: (a) $d_{W}=6$ nm, (b) $d_{W}=12$ nm, (c) $d_{W}=24$ nm and (d) $d_{W}=36$ nm. The barrier width is fixed in all cases to $d_B=6$ nm. As we can notice for $d_W=6$ nm the DOS is accumulated around 25 meV with two symmetric peaks. If we increase the well width to $d_W=12$ nm the accumulation of the DOS is now taking place at around 17 meV, however the peaks are no longer symmetrical. If we further increase the well width to 24 nm the DOS is accumulated around 12 meV and for 36 nm we can see a single peak close to the energy at which Fano resonances take place. Thus, we are corroborating that the improvement of the thermoelectric properties is related to the accumulation of electron states. In the present case of single barrier, the electron states accumulate in the energy region at which Fano resonances are preponderant.

**Figure 9:**
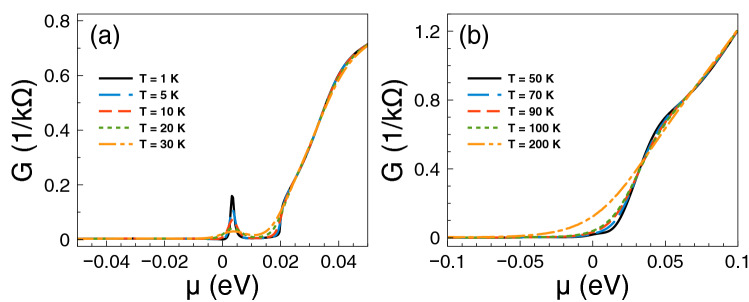
Conductance function versus chemical potential for gapless bilayer graphene double barriers at different temperatures: (**a**) from 1 K to 30 K and (**b**) from 50 to 200 K. The double barriers structural parameters are $d_{B}=d_W=6$ nm and $V_{0}=50$ meV. As we are dealing with symmetric barriers $d_{B1}=d_{B2}=d_B$. At low temperatures it is possible to see the contribution of Breit–Wigner resonances directly on the conductance as a peak as well as how this peak evolves as the temperature rises.

**Figure 10:**
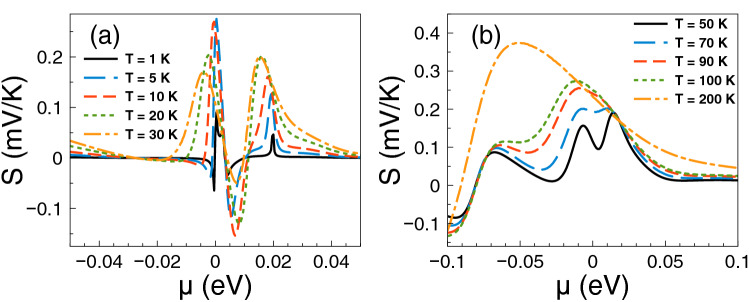
The Seebeck coefficient versus chemical potential for gapless bilayer graphene double barriers at different temperatures: (**a**) from 1 to 30 K and (**b**) from 50 to 200 K. The double barriers structural parameters are the same as in Fig. 9. Here, at low temperatures the charge neutrality point and the Breit–Wigner resonances shape the Seebeck coefficient characteristics, while the Fano resonances energy region become preponderant as the temperature rises, merging with the charge neutrality point and the Breit–Wigner resonances as as single broad peak at high temperatures.

### Bilayer graphene double barriers

Now, it is time to analyze the case of gapless double barriers. Throughout this subsection we will deal with symmetric barriers, that is, the height of the barriers is the same as well as the widths of the barriers and the well. Depending on the structural parameters of the double barriers the transport properties can present energy regions in which Breit–Wigner, Fano and hybrid resonances contribute predominantly^38^.

In first place we will analyze the transport and thermoelectrics of narrow width double barriers. For these barriers Breit–Wigner and Fano resonances shape the transport and thermoelectric properties^38^. In Fig. 9 we show the conductance versus chemical potential at: (a) low temperatures and (b) high temperatures. The width of the barriers-well and the height of the barriers is the same as in the case of single barriers, 6 nm and 50 meV, respectively. As we can see the mentioned resonances are manifested in the conductance. The Breit–Wigner and Fano resonances result in a peak and a sudden rise in the conductance at 5 meV and 20 meV, respectively. The peak and the sudden rise are well-defined at low temperatures and as the temperature rises they systematically soften till finally disappear. Furthermore, at high temperatures the conductance shows an monotonic increasing with the chemical potential as well as an important participation of holes.

After analyzing the fundamental characteristics of the conductance curves of gapless double barriers, we proceed to present the results of the thermoelectric properties. Specifically, the Seebeck coefficient results are shown in Fig. 10. The parameters are the same as in Fig. 9. At very low temperatures we can see the contribution of the charge neutrality point, the Breit–Wigner and Fano resonances. The charge neutrality point as usual results in two asymmetric peaks with negative (holes) and positive (electrons) Seebeck coefficient values. These peaks are related to the change of slope of the conductance at the charge neutrality point. Something similar happens with the peak associated to Breit–Wigner resonances, it results in two asymmetric peaks in the Seebeck coefficient with the positive peak at lower energy than the negative one. The Fano resonances sudden rise contributes with a positive Seebeck coefficient peak. This peak is not as prominent as the peaks associated to the charge neutrality point and the Breit–Wigner resonances. As in the case of single barriers the temperature effects are quite relevant. As the temperature rises the positive peaks of the charge neutrality point and the Breit–Wigner resonances merge as a single peak, being one of the main contributions to the Seebeck coefficient in practically all temperatures assessed. In particular, it dominates the Seebeck coefficient characteristics at low temperature, reaching a maximum of 270 $\mu$V/K at 10 K. The peaks with negative Seebeck coefficient values diminish systematically as the temperature rises, being irrelevant after 20-30 K. At higher temperatures (50 K - 70 K) the peak associated to Fano resonances dominates the Seebeck coefficient, however, at temperatures above 90 K it ends up merging with the peak of the charge neutrality point and the Breit–Wigner resonances in a broad peak in the holes energy region.

**Figure 11:**
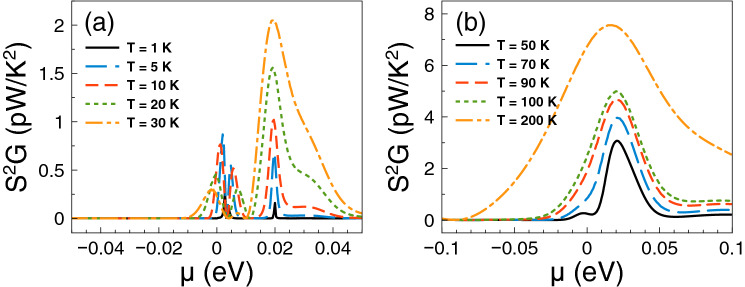
Power factor versus chemical potential for gapless bilayer graphene double barriers at different temperatures: (**a**) from 1 to 30 K and (**b**) from 50 to 200 K. The double barriers structural parameters are the same as in Fig. 9. In this case, at low temperatures, there is a competency between the charge neutrality point and Breit–Wigner resonance with the Fano resonances energy region. However, as the temperature increases the Fano resonances dominate the power factor characteristics.

With the calculation of the conductance and the Seebeck coefficient we can obtain straightforwardly the power factor $S^{2}G$. The corresponding results of the power factor are shown in Fig. 11. As we can notice there is a significant difference with respect to single barriers. The contribution of the Breit–Wigner resonances determine the power factor characteristics at low temperatures. In fact, the power factor presents two asymmetric peaks around the charge neutrality point. One of these peaks reaches a maximum value of 0.8 pW/K$^2$ at 5 K. For temperatures equal or above 10 K the main contribution comes from Fano resonances with a peak that increases and broadens monotonically as the temperature rises. At high temperature this peak becomes an order of magnitude (8 pW/K$^2$) larger than its low temperature counterpart.

**Figure 12:**
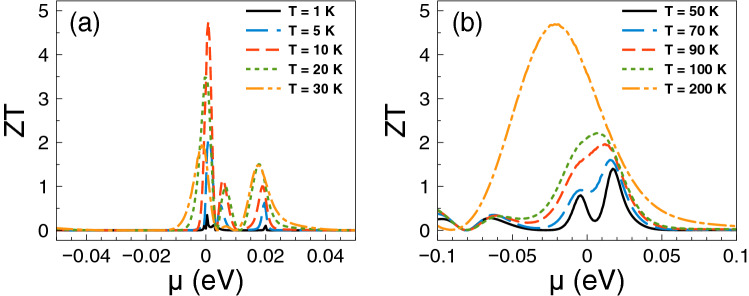
*ZT* versus chemical potential for gapless bilayer graphene double barriers at different temperatures: (**a**) The range from 1 to 30 K and (**b**) the range from 50 to 200 K. The double barriers structural parameters are the same as in the preceding figures. As in the case of the Seebeck coefficient, the charge neutrality point and Breit–Wigner resonances dominate the *ZT* characteristics at low temperatures, while the Fano resonances become preponderant as the temperature rises, merging with the charge neutrality point and Breit–Wigner resonances as a single broad peak at high temperatures.

**Figure 13:**
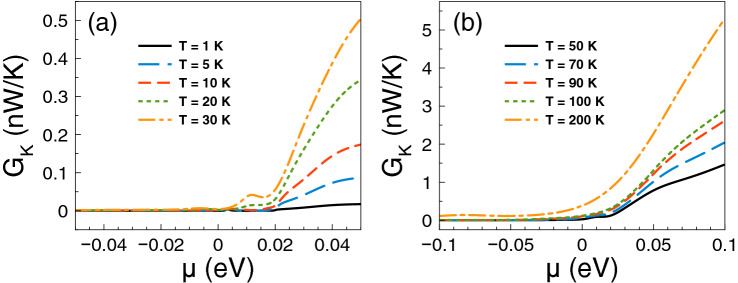
Electronic thermal conductance versus chemical potential for gapless bilayer graphene double barriers at different temperatures: (**a**) from 1 to 30 K and (**b**) from 50 to 200 K. The double barrier structural parameters are the same as in the preceding figures. Here, it is important to remark that $G_K/(GT)$ is not the Lorenz number due to the nanostructuration effects. This results in a reduction of *ZT* about an order of magnitude with respect to strained single layer graphene^54^. The outcomes of $G_K/(GT)$ and $S^2$ can be found in the supplementary information.

**Figure 14:**
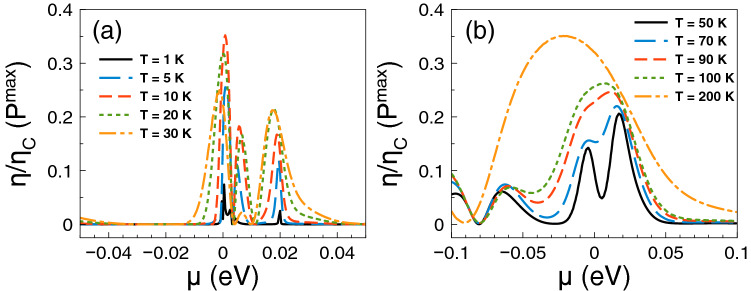
Efficiency at maximum power versus chemical potential for gapless bilayer graphene single barriers at different temperatures: (**a**) from 1 to 30 K and (**b**) from 50 to 200 K. The double barrier structural parameters are the same as in the preceding figures. The maximum efficiency is reached at the temperatures at which *ZT* is maximum as well.

**Figure 15:**
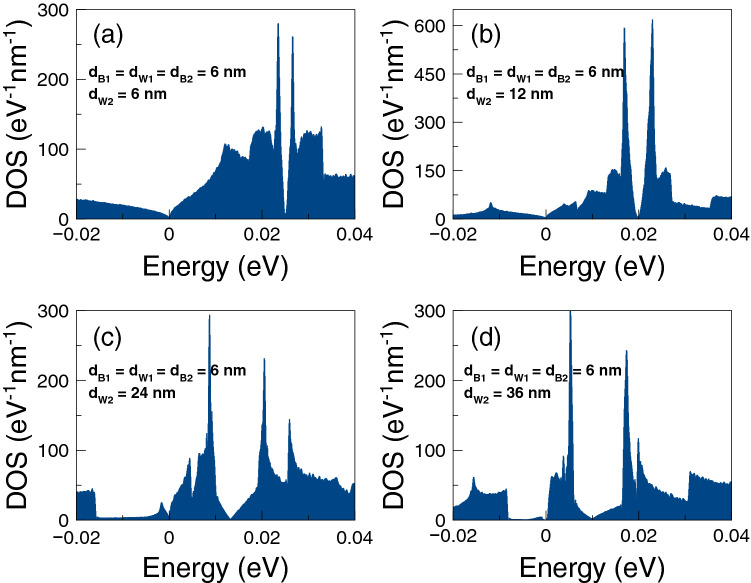
Density of states versus the energy for gapless bilayer graphene double barriers of $d_B=d_W=6$ nm. Here, the width of the second well $d_{W2}$ is varied to mimic the conditions for double barriers. The height of the barriers is $V_{0}=50$ meV. In this case, as $d_{W2}$ increases the electron states accumulate around the Breit–Wigner and Fano resonances energy regions.

**Figure 16:**
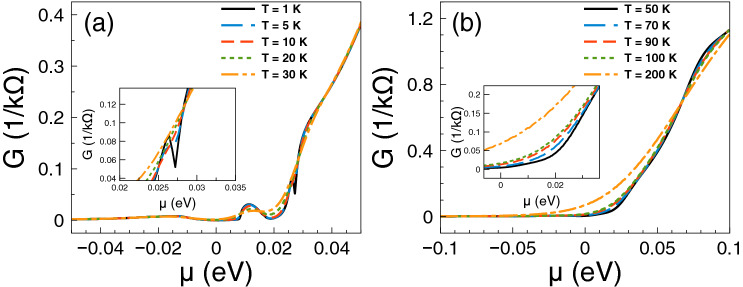
The same as Fig. 9, but for $d_B=d_W=10$ nm. Here, in addition to the contribution of the Breit–Wigner resonances we can see the contribution of the so-called hybrid resonances at about 26 meV, see the inset in (a).

**Figure 17:**
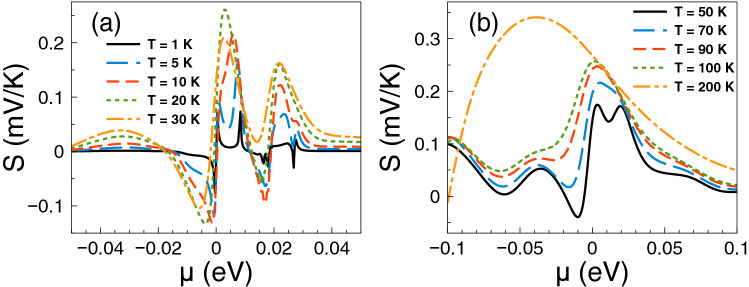
The same as Fig. 10, but for $d_B=d_W=10$ nm. As it is noted, at low temperatures the charge neutrality point and the Breit–Wigner resonances shape the Seebeck coefficient characteristics, while the hybrid resonances become preponderant as the temperature rises, merging with the charge neutrality point and the Breit–Wigner resonances as as single broad peak at high temperatures.

**Figure 18:**
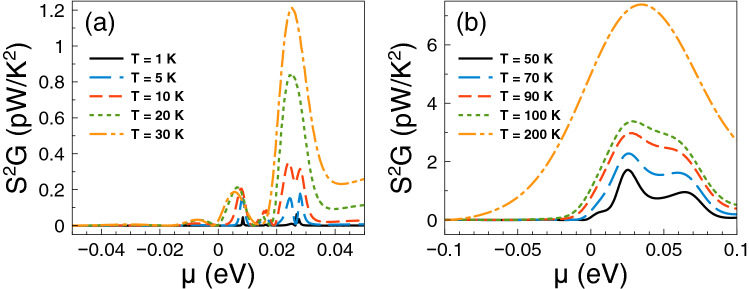
The same as Fig. 11, but for $d_B=d_W=10$ nm. In this case, hybrid resonances dominate the power factor characteristics after $T=5$ K. In fact, hybrid resonances contribute with two symmetric peaks at low temperatures, merging as a single broad peak as the temperature rises.

**Figure 19:**
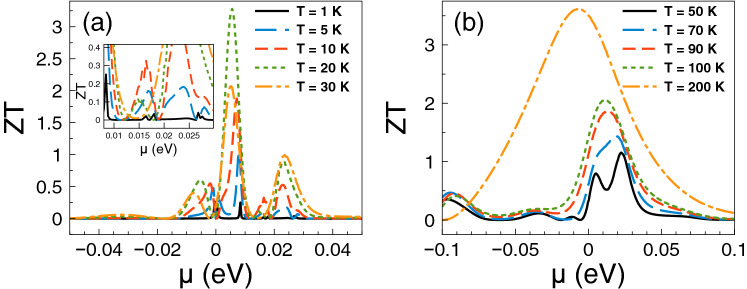
The same as Fig. 12, but for $d_B=d_W=10$ nm. Here, the charge neutrality point and Breit–Wigner resonances shape the *ZT* characteristics at low temperatures, while the contribution of hybrid resonances become preponderant at the temperature rises, merging as a single broad peak at high temperatures.

**Figure 20:**
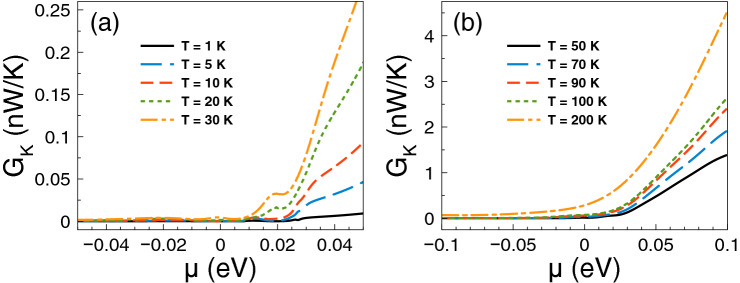
The same as Fig. 13, but for $d_B=d_W=10$ nm. As in the other barrier cases, the ratio of $G_K$ to *GT* is not the Lorenz number. So, the contribution of $S^2$ to *ZT* is diminished by $G_K/(GT)$, resulting in *ZT*s an order of magnitude lower than in strained single layer graphene^54^. The results of $G_K/(GT)$ and $S^2$ can be found in the supplementary information.

**Figure 21:**
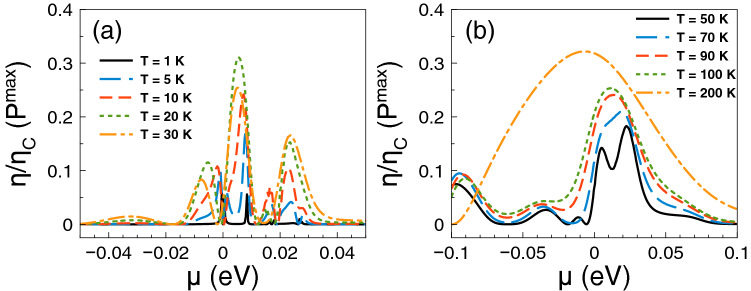
The same as Fig. 14, but for $d_B=d_W=10$ nm. In this case, we also have more options for an acceptable output power and a good efficiency owing to the different resonances in the double barrier structure.

**Figure 22:**
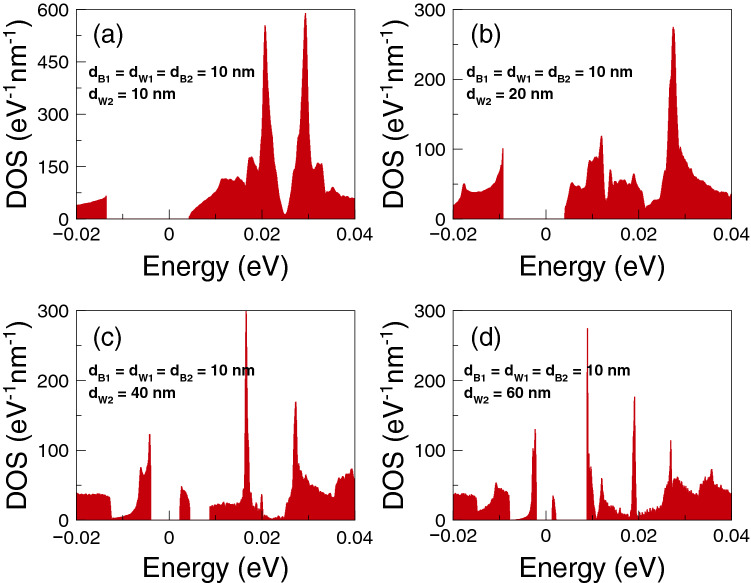
The same as Fig. 15, but for $d_B=d_W=10$ nm. Here, the width of the second well takes the values: (**a**) $d_{W2}=10$ nm, (**b**) $d_{W2}=20$ nm, (**c**) $d_{W2}=40$ nm and (**d**) $d_{W2}=60$ nm. As $d_{W2}$ increases the electron states accumulate in the Breit–Wigner and hybrid resonances energy regions.

In the case of *ZT*, we find that its dependence with the chemical potential and temperature is similar to that of the Seebeck coefficient. This in great extent to the partial cancellation of the contribution of the electrical and thermal conductances, compare Figs. 9 and 13. The specific results are shown in Fig. 12. As we can notice the peaks associated to the charge neutrality point and Breit–Wigner resonances dominate *ZT* at low temperatures. In fact, the peak close to the charge neutrality point reaches a maximum of 5 at 10 K. For temperatures above 50 K, *ZT* is dominated by the peak associated to Fano resonances, however as the temperature rises this peak is combined with the low energy one associated to the charge neutrality point and the Breit–Wigner resonances, merging in a single and broad peak. *ZT* reaches values of 2 and 4.5 for 100 K and 200 K, respectively. These values for *ZT* at low and high temperatures are significantly larger than those reported for conventional semiconductors^67,68^. It is also important to mention that the mentioned values of *ZT* at low temperatures are in great extent owe to the presence of Breit–Wigner resonances. To contrast see the results of *ZT* for single barriers (Fig. 5) in which there are no Breit–Wigner resonances. As in the case of single barriers, *ZT* is not as high as in the case of strained single layer graphene with a spin flipper^54^. The dependence of $G_K/(GT)$ with the chemical potential and temperature as a result of the nanostructuration diminishes the contribution of $S^2$, resulting in values of *ZT*an order of magnitude lower than in strained single layer graphene^54^. The corresponding results of $G_K/(GT)$ and $S^2$ for double barriers can be found in the supplementary information. In addition, a comparison of the thermoelectric response of bilayer graphene with double barriers and without them is included in the supplementary information. From the comparison, we can see that the nanostructuration and its associated effects (resonances) are quite relevant to improve the thermoelectric response in bilayer graphene.

Regarding the maximum power and the efficiency at maximum power, we find a more intricate dynamic than in single barriers due to the different resonances in play. As in the case of single barriers, we can obtain the maximum power readily by weighing the power factor results by 1/4 and $(\Delta T)^2$. In Fig. 14 we show the efficiency at maximum power as a function of the chemical potential at different temperatures as indicated. Here, we have more options for a reasonable power and a good efficiency. For instance, at low temperatures, we can choose $T=10$ K with $P^{max} \approx 0.2$ pW$(\Delta T)^2$/K$^2$ and $\eta \approx 0.35 \eta _c$, taking place around the energy region of the Breit–Wigner resonances and the charge neutrality point. We can also sacrifice efficiency gaining output power by choosing $T=30$ K with $P^{max} \approx 0.5$ pW$(\Delta T)^2$/K$^2$ and $\eta \approx 0.22\eta _c$, happening around the Fano resonances energy region. At high temperatures, we can also have a good trade off between output power and efficiency. Specifically, at $T=100$ K and $\mu \approx 20$ meV, we obtain $P^{max} \approx 1.25$ pW$(\Delta T)^2$/K$^2$ and $\eta \approx 0.24\eta _c$. Likewise, at $T=200$ K and $\mu \approx 20$ meV, we can increase the output power up to $P^{max} \approx 1.95$ pW$(\Delta T)^2$/K$^2$, while maintaining an acceptable efficiency $\eta \approx 0.25\eta _c$. Here, it is also important to mention that as in the case of single barriers the maximum power is more than an order of magnitude higher than the one reported in strained single layer graphene^54^.

To end with these specific double barriers, we will analyze if the accumulation of electron states is involved in the improvement of the thermoelectric properties. In Fig. 15 we show the results for the DOS as a function of the energy. In this case the unit-cell of the one dimensional periodic structure is composed of two barriers and two wells. The width of the second well is enlarged in order to mimic the double barriers conditions. In particular, the widths considered for the second well are: (a) 6 nm, (b) 12 nm, (c) 24 nm and (d) 36 nm. The other structural parameters are the same as for the transport and thermoelectric properties $d_{B1}=d_{W1}=d_{B2}=6$ nm and $V_0=50$ meV. In the case of $d_{W2}=6$ nm the DOS is the same as for single barriers. This is because the one dimensional periodic systems are the same, the only difference is the size of the unit-cell. Once the width of the second well is increased the DOS is redistributed. For instance, in the case of $d_{W2}=12$ nm the electron states are accumulated around 20 meV, with two almost symmetrical peaks. For $d_{W2}=24$ nm the DOS presents a minimum close to 15 meV and two leading peaks at 10 meV and 20 meV. For $d_{W2}=36$ nm the leading peaks have moved to 5 meV and 18 meV, which corresponds to the energy regions where Breit–Wigner and Fano resonances are preponderant. So, these findings indicate that the accumulation of electron states is directly implicated in the improvement of the thermoelectric properties.

If we change the structural parameters other resonances come into play. In the case of double barriers of 10 nm, the interplay between Fano resonances and resonant states of the quantum well region give rise to the so-called hybrid resonances^38^. In fact, in Fig. 16 we can see that the Breit–Wigner and Hybrid resonances shape the conductance curves profile. The hybrid resonances give rise to a peak followed by a minimum at about 27 meV, while Breit–Wigner resonances result in a broad peak centered about 12 meV. The latter peak has shifted to higher energies with respect to the corresponding one of 6 nm double barriers. The conductance also presents a change of slope at the charge neutrality point. Furthermore, we can notice that temperature effects are quite relevant for these barriers. The mentioned peaks soften as temperature rises till eventually disappear. At high temperatures the conductance is a monotonic increasing function of the chemical potential. All these conductance characteristics shape the Seebeck coefficient as shown in Fig. 17. In fact, the charge neutrality point, the Breit–Wigner and Fano resonances result in two alternate peaks each. The positive peaks associated to the charge neutrality point and the Breit–Wigner resonances combine in a single peak as the temperature increases, being the main contribution to the Seebeck coefficient up to 30 K. This peak reaches a maximum of 260 $\mu$V/K at 20 K. The negative peaks diminish as the temperature rises, being negligible after 50 K. The peak associated to hybrid resonances contributes equally at 50 K than the one related to the charge neutrality point and the Breit–Wigner resonances. For higher temperatures these two peaks merge as a single one, shifting to lower energies and representing the main contribution to the Seebeck coefficient.

Regarding the power factor, the main contributions come from Breit–Wigner and hybrid resonances. The specific power factor results are shown in Fig. 18. Up to 5 K Breit–Wigner and hybrid resonances contribute equally. For higher temperatures the peak associated to hybrid resonances increases and broadens becoming the dominant contribution. The power factor reaches values of 3.5 and 7.5 pW/K$^2$ for 200 and 300 K respectively. Here, the conductance is the key factor that potentiate hybrid resonances as the dominant contribution of the power factor. In fact, the conductance increases significantly at the energy region in which hybrid resonances become preponderant. In the case of *ZT* we find multiple peaks associated to the charge neutrality point, Breit–Wigner and hybrid resonances as shown in Fig. 19. At low temperatures, up to 30 K, the peak related to Breit–Wigner resonances dominates *ZT* reaching a maximum of 3.5 at 20 K. The peak associated to hybrid resonances becomes dominant at 50 K, reaching a *ZT* value of 1. For higher temperature the leading peaks merge as a single one that broadens with temperature. This peak becomes the main contribution to *ZT* at high temperatures, reaching values of 2 and 3.5 for 200 and 300 K, respectively. This dynamic of *ZT* obeys the partial cancellation of the electrical and thermal conductances. As we can see in Fig. 20 the thermal conductance has a similar dependence with the chemical potential and temperature as the electrical one. So, the Seebeck coefficient is what predominately shape the *ZT* characteristics. However, $G_K/(GT)$ is not at all the Lorenz number as in the case of strained single layer graphene^54^. Thus, the values of *ZT* are an order of magnitude lower than in strained single layer graphene^54^. More details about $G_K/(GT)$ and $S^2$ for these double barriers are shown in the supplementary information. As in the other cases, the comparison of bilayer graphene with and without barriers are included in the supplementary information. We also have different options to obtain a good trade off between output power and efficiency as shown in Fig. 21. For instance, we can take advantage of the hybrid resonances energy region ($\mu =25$ meV) to obtain an output power of $P^{max}=0.2$ pW$(\Delta T)^2$/K$^2$ as well as an efficiency of $\eta \approx 0.15\eta _c$. At higher temperatures we can also obtain a good trade off between output power and efficiency. In particular, at $T=100$ K and $\mu \approx 30$ meV, the output power and the efficiency reach values of $P^{max}=0.85$ pW$(\Delta T)^2$/K$^2$ and $\eta \approx 0.2\eta _c$, respectively. Finally, as in the other barrier cases, we corroborate that the accumulation of the electron states is directly implicated in the improvement of the thermoelectric properties. The specific results of the DOS are shown in Fig. 22.

## Conclusions

In summary, we have analyzed the temperature dependence of the transport and thermoelectric properties of gapless bilayer graphene single and double barrier structures. In particular, we have assessed the impact of the system resonances on the thermoelectric response. As the phonon thermal transport is negligible in barrier structure devices, we focus our study on the electronic thermoelectric response. We have described the charge carriers in bilayer graphene as massive chiral particles through a four-band Hamiltonian. We have used a modified version of the hybrid matrix method as well as the Landauer–Büttiker formalism to obtain the transmission and thermoelectric properties, respectively. We found that the charge neutrality point and the system resonances shape the thermoelectric response. In the case of single barriers, the charge neutrality point dominates the thermoelectric response at low temperatures, while the Fano resonances become preponderant as the temperature rises. These temperature regimes are clearly seen in the Seebeck coefficient, *ZT* and $\eta (P^{max})$, with the crossover presented at 5 K. These three quantities reach its maximum value at 50 K, with 250 $\mu$V/K, 1.8 and $0.22\eta _c$ as maximum values, respectively. For the power factor, the Fano resonances dominate at all temperatures. Specifically, the power factor has a peak at the energy region in which the Fano resonances take place. The peak is sharp at low temperatures and gets wider as the temperature increases. The power factor also presents a monotonic increase with temperature. In the case of double barriers, Breit–Wigner resonances dominate at low temperatures, while Fano and hybrid resonances become preponderant as the temperature increases. Depending on the structural parameters, we can have Breit–Wigner and Fano resonances or Breit–Wigner and hybrid resonances in play. For temperatures below 50 K the Seebeck coefficient, *ZT* and $\eta (P^{max})$ are determined by Breit–Wigner resonances, reaching values of 270 $\mu$V/K, 4.8 and $0.35\eta _c$, respectively. For temperatures above 50 K, Fano and hybrid resonances govern the characteristics of the Seebeck coefficient, *ZT* and $\eta (P^{max})$, with values similar as in the low-temperature regime. For the power factor, the temperature regime crossover takes place at 5 K, with the Fano and hybrid resonances dominant for most temperatures. For both single and double barriers the system resonances also allow us to optimize the output power and efficiency. In addition, we computed the density of states for both single and double barriers, finding that the electron states accumulate in the energy regions in which the thermoelectric response is improved. So, our findings support the idea that bilayer graphene barrier structures can be a based system to improve the performance of thermoelectric devices by exploiting the system resonances as well as by reducing the phonon thermal transport by supporting and encapsulating the graphene layers.

## Supplementary Information

Supplementary Information.

## Data Availability

The data that support the findings of this study are available from the corresponding author upon reasonable request.
