# Supplementary material for: Enhancement of the thermoelectric properties in bilayer graphene structures induced by Fano resonances
Source: Sci Rep. 2021 Jul 6;11:13872. doi: 10.1038/s41598-021-93220-w (PMC8260707; doi:10.1038/s41598-021-93220-w)
Supplement: Supplementary file 1 — Supplementary Information. [file 41598_2021_93220_MOESM1_ESM.pdf]

Supplementary Information

Enhancement of the thermoelectric properties in bilayer graphene structures induced by Fano resonances

J. A. Briones-Torres[1], R. Pérez-Álvarez[1,2], S. Molina-Valdovinos[1] and I. Rodríguez-Vargas[1]

[1] *Unidad Académica de Ciencia y Tecnología de la Luz y la Materia, Universidad Autónoma de Zacatecas, Carretera Zacatecas-Guadalajara Km. 6, Ejido La Escondida, 98160 Zacatecas, Zacatecas, Mexico.*

[2] *Centro de Investigación en Ciencias, Universidad Autónoma del Estado de Morelos, Av. Universidad 1001 Col. Chamilpa, 62209 Cuernavaca, Morelos, Mexico.*

S1. Single barriers: $G_K/(GT)$, S^2 and comparison of the thermoelectric response with and without barriers

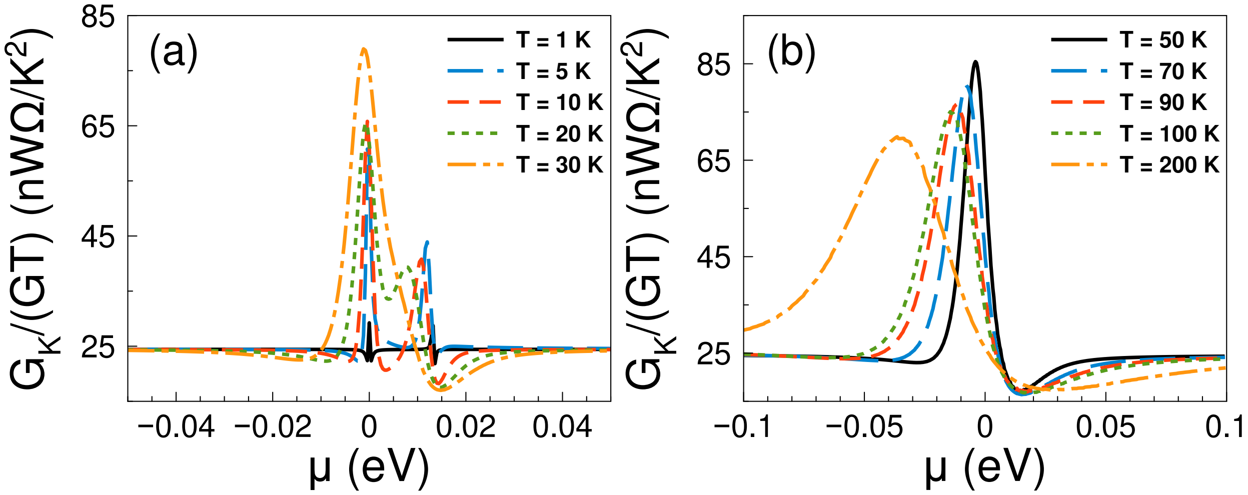

Fig. S1: $G_K/(GT)$ versus chemical potential for single barriers at different temperatures as indicated. The single barrier structural parameters are $d_B = 6$ nm and $V_0 = 50$ meV. Here, $G_K/(GT)$ is not the Lorenz number as in the case of strained single layer graphene.[1]

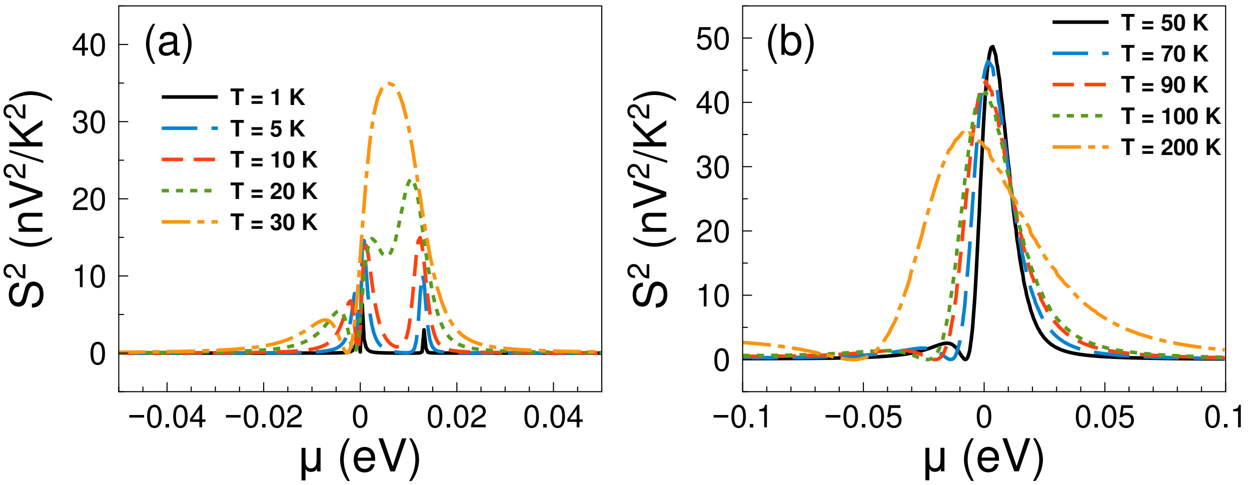

Fig. S2: S^2 versus chemical potential for single barriers at different temperatures as indicated. The single barrier structural parameters are the same as in Fig. S1. Here, S^2 is diminished by $G_K/(GT)$, resulting in figures of merit an order of magnitude lower than in the case of strained single layer graphene.[1]

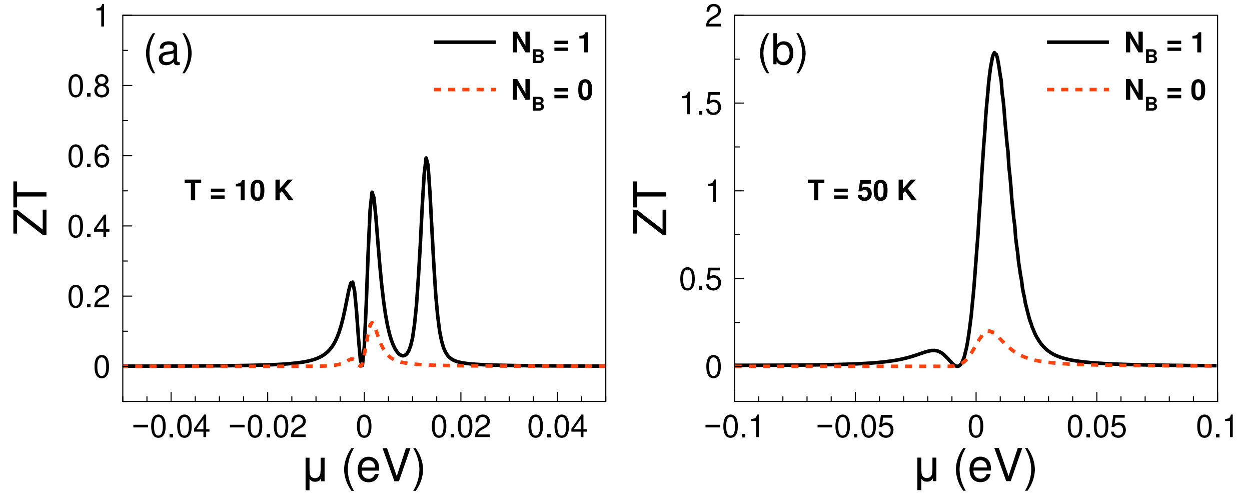

Fig. S3: Comparison of the figure of merit with and without barriers at (a) $T = 10$ K and (b) $T = 50$ K. The structural parameters are the same as in the preceding figures. As we can notice the thermoelectric response is enhanced significantly by the effect of the single barriers.

S2. Double barriers $d_B = d_W = 6$ nm: $G_K/(GT)$, S^2 and comparison of the thermoelectric response with and without barriers

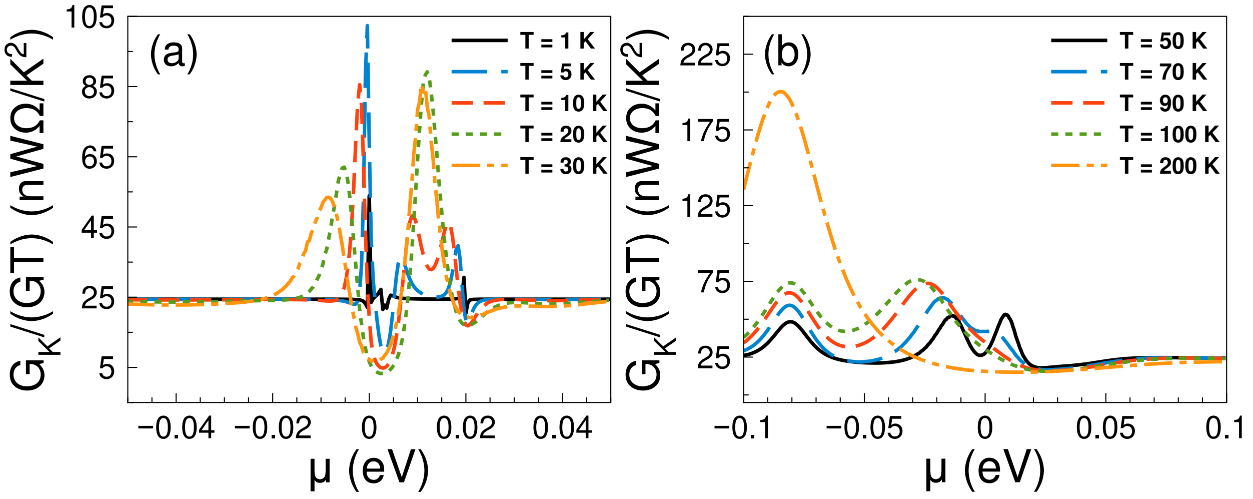

Fig. S4: $G_K/(GT)$ versus chemical potential for double barriers at different temperatures as indicated. The double barrier structural parameters are $d_B = d_W = 6$ nm and $V_0 = 50$ meV. Here, $G_K/(GT)$ is not the Lorenz number as in the case of strained single layer graphene.[1]

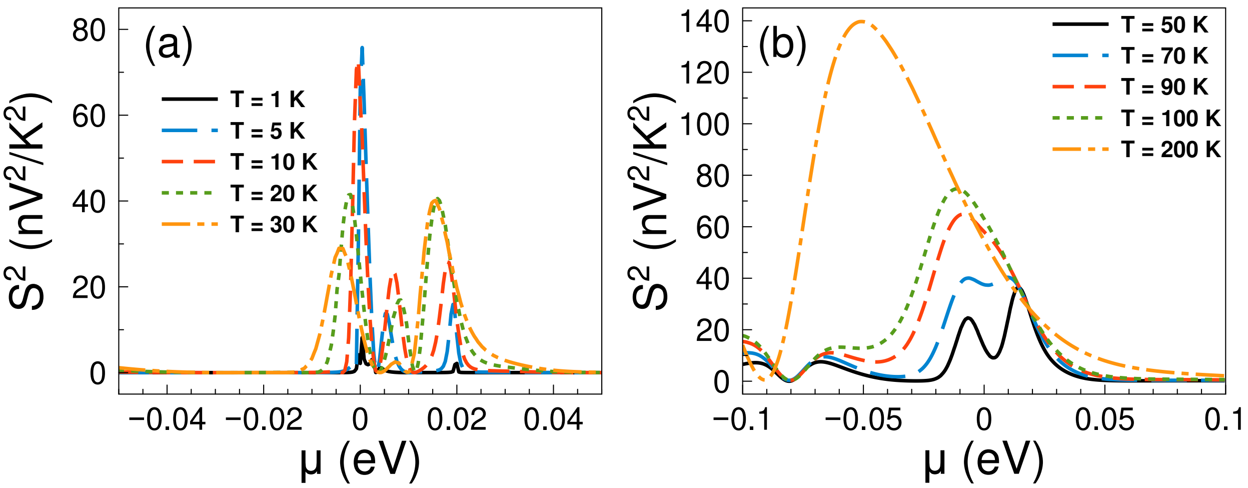

Fig. S5: S^2 versus chemical potential for single barriers at different temperatures as indicated. The double barrier structural parameters are the same as in Fig. S4. Here, S^2 is diminished by $G_K/(GT)$, resulting in figures of merit an order of magnitude lower than in the case of strained single layer graphene.[1]

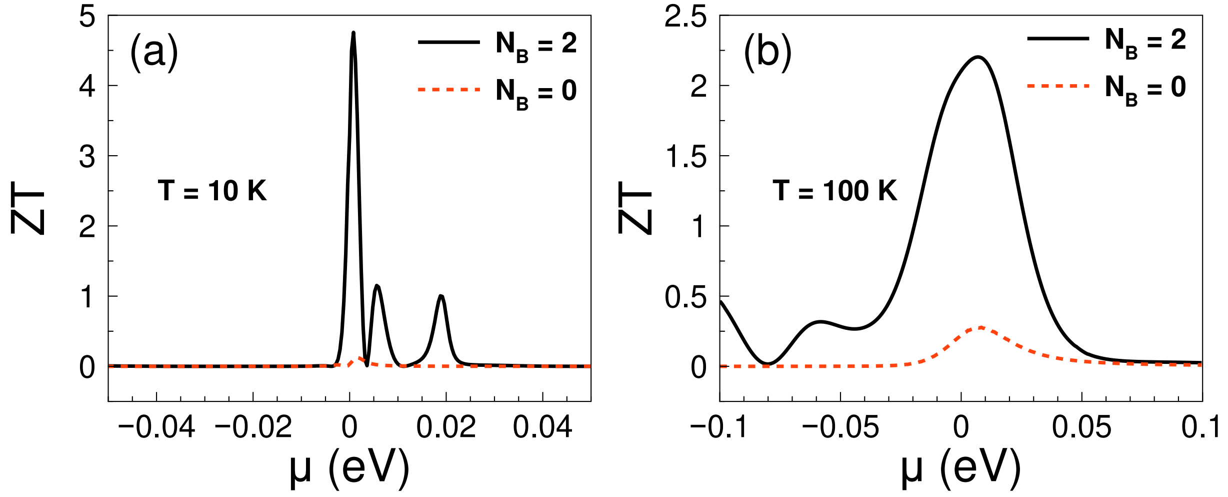

Fig. S6: Comparison of the figure of merit with and without barriers at (a) $T = 10$ K and (b) $T = 100$ K. The structural parameters are the same as in the preceding figures. As we can notice the thermoelectric response is enhanced significantly by the effect of the double barriers.

S3. Double barriers $d_B = d_W = 10$ nm: $G_K/(GT)$, S^2 and comparison of the thermoelectric response with and without barriers

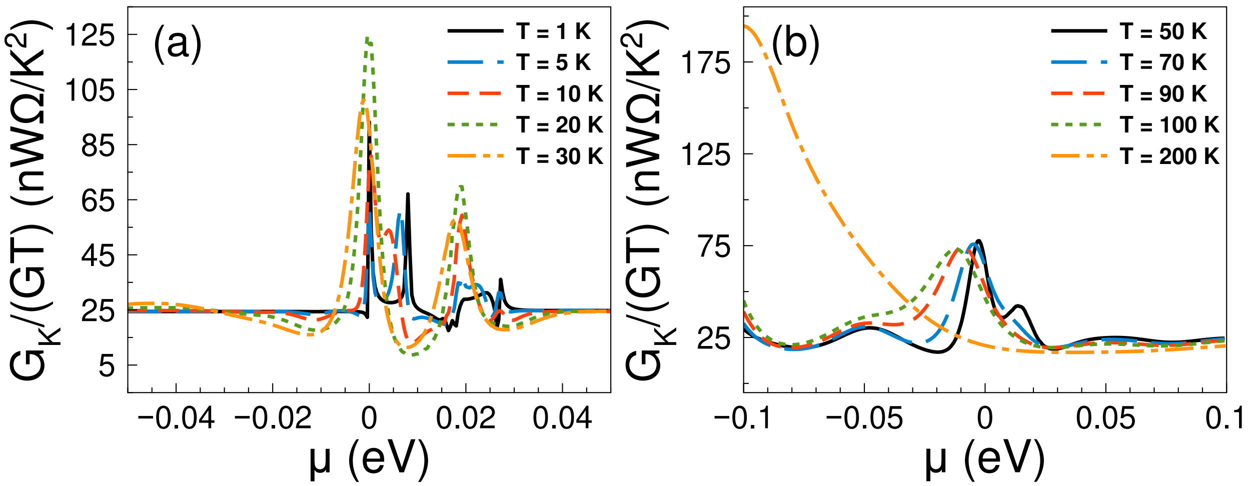

Fig. S7: $G_K/(GT)$ versus chemical potential for double barriers at different temperatures as indicated. The double barrier structural parameters are $d_B = d_W = 10$ nm and $V_0 = 50$ meV. Here, $G_K/(GT)$ is not the Lorenz number as in the case of strained single layer graphene.[1]

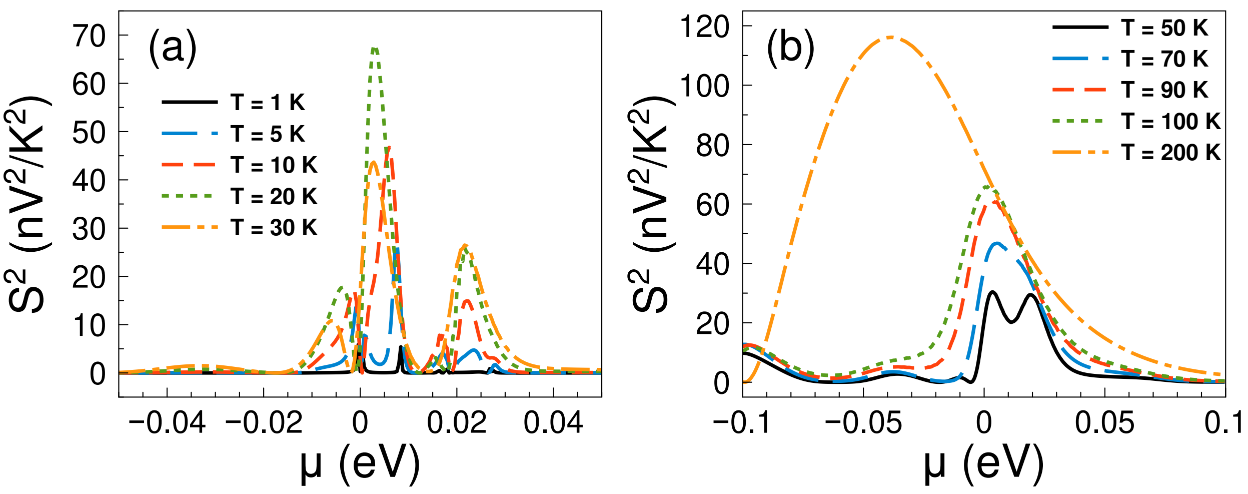

Fig. S8: S^2 versus chemical potential for single barriers at different temperatures as indicated. The double barrier structural parameters are the same as in Fig. S7. Here, S^2 is diminished by $G_K/(GT)$, resulting in figures of merit an order of magnitude lower than in the case of strained single layer graphene.[1]

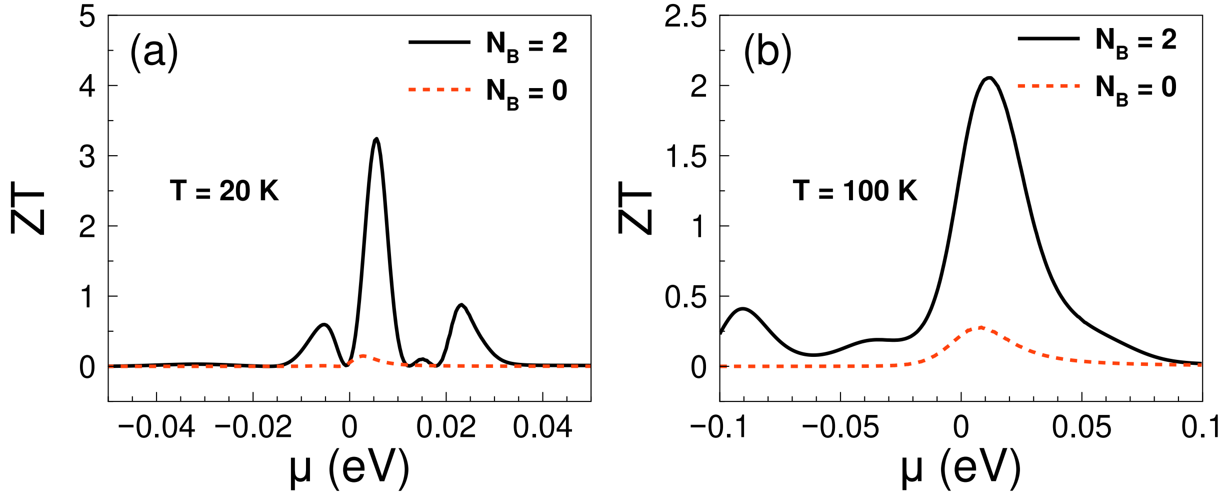

Fig. S9: Comparison of the figure of merit with and without barriers at (a) $T = 20$ K and (b) $T = 100$ K. The structural parameters are the same as in the preceding figures. As we can notice the thermoelectric response is enhanced significantly by the effect of the double barriers.

Supplementary References

1. Mani, A, Pal, S. & Benjamin, C., Designing a highly efficient graphene quantum spin heat engine, *Sci. Rep.* **9**, 6018 (2019).
